# Supplementary material for: High annual-cycle repeatability suggests low flexibility to environmental changes in a near-threatened migratory shorebird
Source: Commun Biol. 2026 May 30;9:736. doi: 10.1038/s42003-026-10371-0 (PMC13222360; doi:10.1038/s42003-026-10371-0)
Supplement: Supplementary file 1 — Supplementary information [file 42003_2026_10371_MOESM1_ESM.pdf]

High annual-cycle repeatability suggests low flexibility to environmental changes in a near-threatened migratory shorebird

Philipp Schwemmer[a*], Marie Donnez[b], Moritz Mercker[c], Stefan Garthe[a], Martin Boschert[d], Heinz Düttmann[e], Jaanus Elts[f], Thomas Fartmann[g,h], Wolfgang Fiedler[i,j], Frédéric Jiguet[k], Steffen Kämpfer[g], Michał Korniluk[l,m], Helmut Kruckenberg[n], Dominik Krupiński[o], Riho Marja[f,p], Markus Piha[q,r], Pierre Rousseau[s], Verena Rupprecht[t], Pierrick Bocher[b]

Supplementary information

Supplement 1:

Table S1: Repeatability index and 95% confidence intervals (CI) for spatial, migration and stopover parameters in curlews during spring and autumn migration.

	Number of obs.	Number of birds	R	95% CI	p-value
**Spatial repeatability**					
Breeding site latitude	89	35	0.997	0.996-0.999	**<0.001**
Breeding site longitude	89	35	0.999	0.999-1	**<0.001**
Wintering site latitude	159	63	0.999	0.997-0.999	**<0.001**
Wintering site longitude	159	63	0.998	0.995-1	**<0.001**

	Spring					Autumn				
	Number of obs.	Number of birds	R	95% CI	p-value	Number of obs.	Number of birds	R	95% CI	p-value
**Migration repeatability**										
Departure date	89	35	0.537	0.184-0.73	**0.02**	159	63	0.307	0.069-0.495	0.053
Arrival date	89	35	0.577	0.297-0.749	**0.002**	159	63	0.362	0.132-0.549	**0.015**
Duration of migration	89	35	0.738	0.511-0.85	**<0.001**	159	63	0.534	0.324-0.683	**<0.001**
Linear distance breeding / wintering site	89	35	0.949	0.894-0.974	**<0.001**	159	63	0.971	0.95-0.982	**<0.001**

Flown distance breeding / wintering site	89	35	0.638	0.423-0.777	**<0.001**	159	63	0.471	0.249-0.63	**0.001**
**Stopover repeatability**										
Mean stopover length	87	35	0.682	0.426-0.825	**<0.001**	143	63	0.462	0.216-0.643	**<0.001**
Total stopover length	89	35	0.723	0.477-0.846	**<0.001**	159	63	0.427	0.249-0.63	**0.001**
Total number of stopovers	89	35	0.52	0.216-0.724	**0.036**	159	63	0.244	0.007-0.435	0.235

Supplement 2:

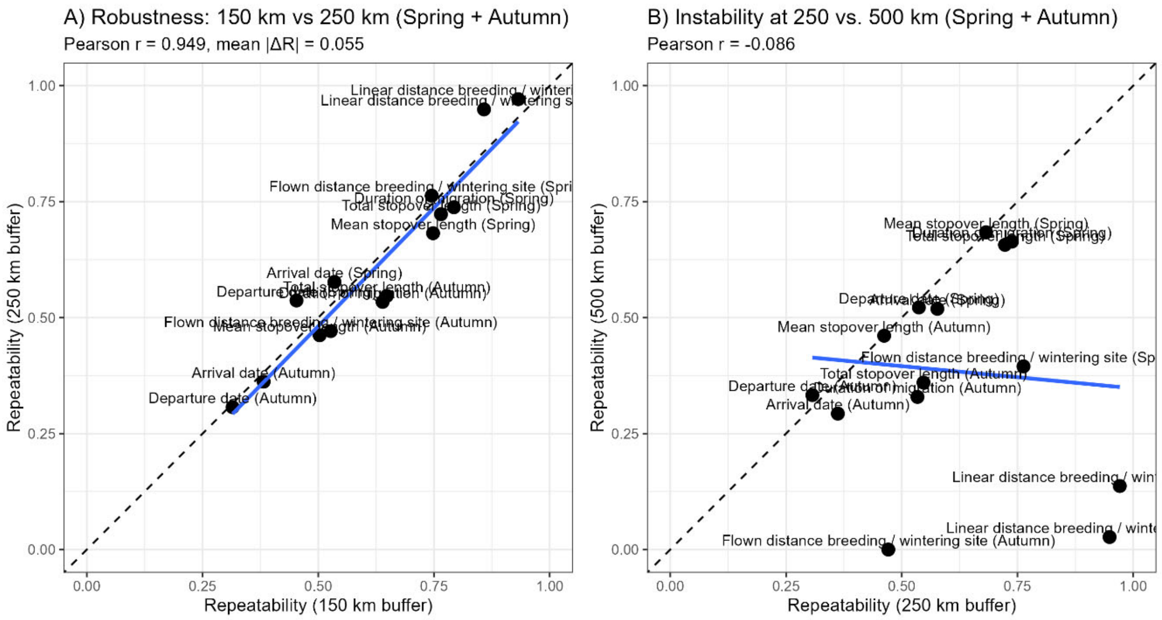

Figure S1: Repeatability estimates were nearly identical among all migration parameters and both seasons when comparing 150 km and 250 km breeding and wintering clusters (see Fig. A above; Pearson correlation = 0.98, mean |ΔR| = 0.035, maximum |ΔR| = 0.105). At a 500 km buffer, the number of spatial clusters decreased markedly. This over-aggregation reduced between-individual variance and led to numerical instability in several models (e.g Breeding area longitude, linear and flown distance breeding / wintering site, respectively), some of which returned degenerate variance components with R ≈ 0 (see Fig. Ba above). Such singular fits are a known phenomenon in mixed-effects models when the estimated random-effect variance approaches zero or random effects become perfectly correlated (Bates et al. 2015). We therefore interpret the 500 km results as unreliable and restrict inference to the stable 150 km and 250 km analyses

Bates D, Mächler M, Bolker B, Walker S (2015) Fitting linear mixed-effect models using lme4. Journal of Statistical Software 67: 1-48. https://doi.org/10.18637/jss.v067.i01

Supplement 3:

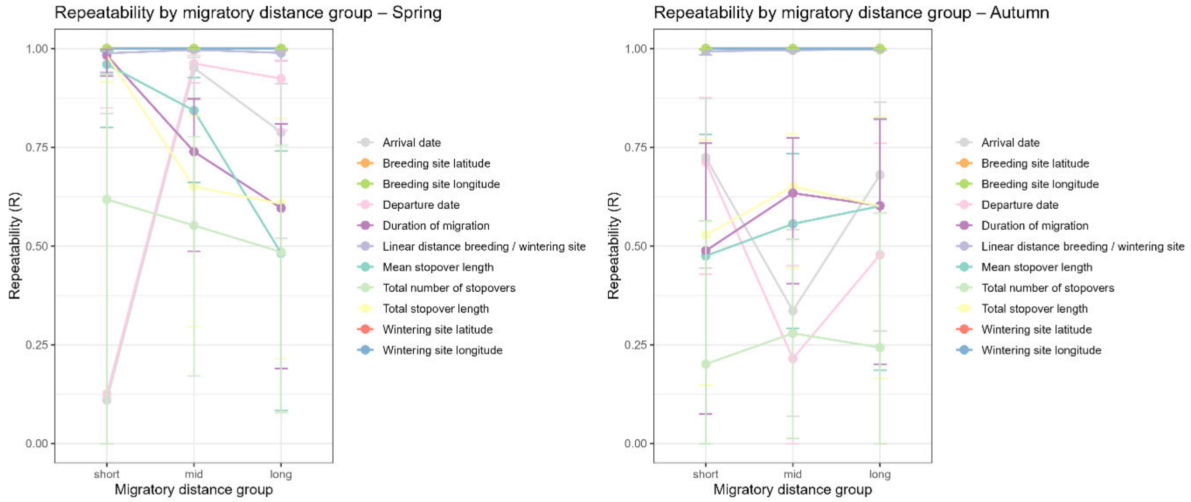

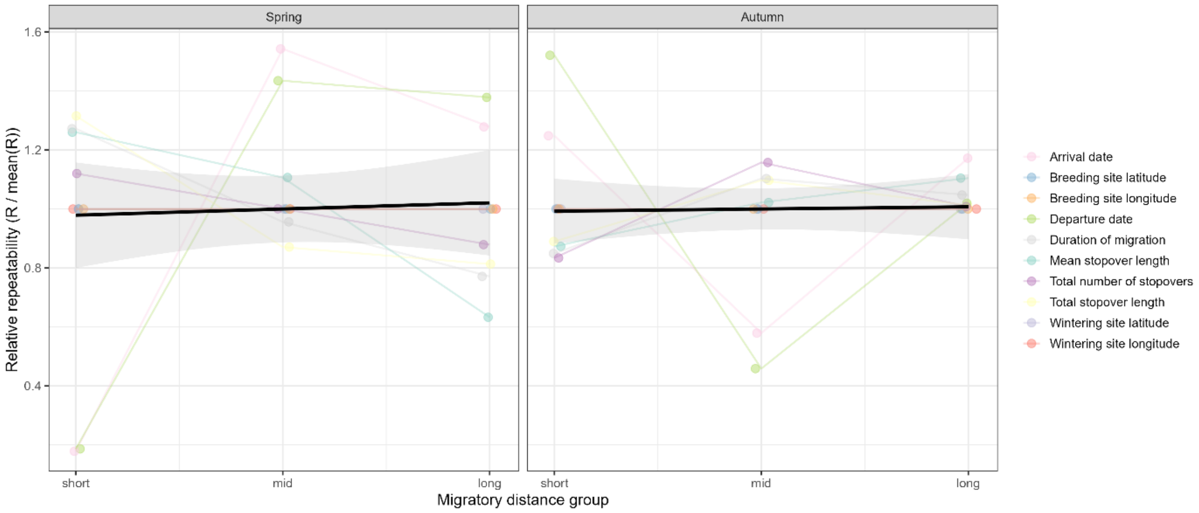

Figure S2 – above: Repeatability values (dots) and 95% confidence intervals (bars) of all parameters of interest split into the groups of short-, mid- and long-distance migrants (according to the quantiles of the migration distances exhibited by the birds) for spring (left) and autumn migration (right).

Figure S2 – below: Re-scaled R values and linear regression (bold line) across all parameters of interest. Grey shaded area: 95% confidence interval of the regression line. For colour codes of the parameters of interest see Figure above.

Supplement 4:

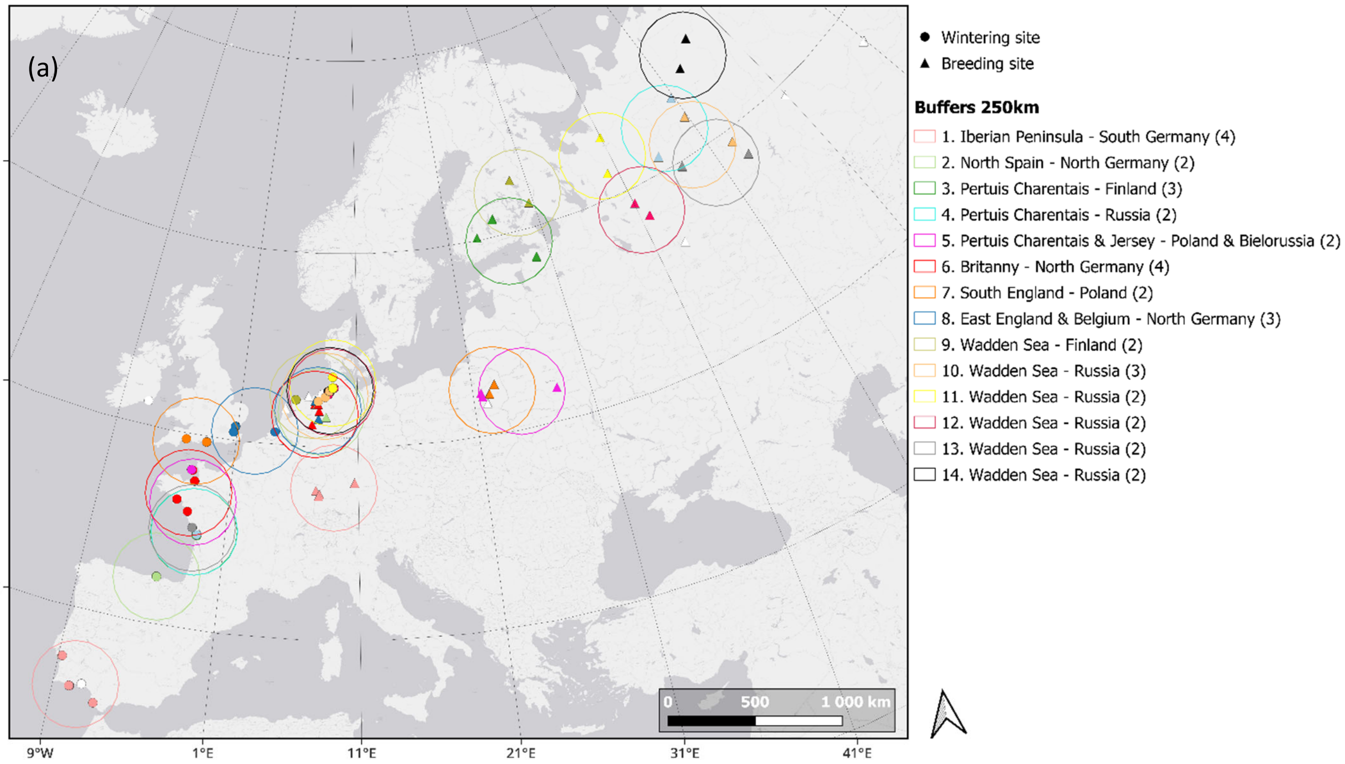

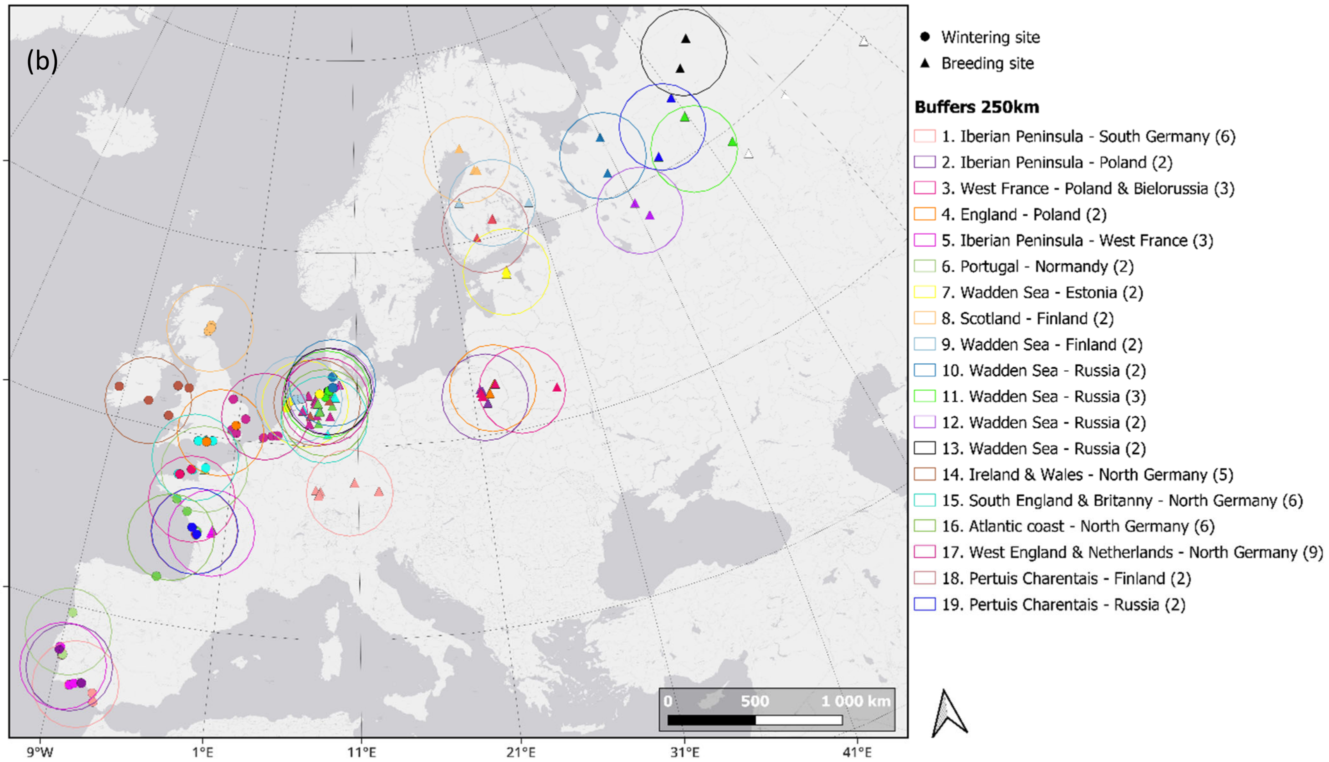

Figure S3: Spatial clusters (250 km buffers) of (a) curlews commuting between the same breeding and wintering sites during spring migration (14 clusters, 35 individuals) and (b) curlews commuting between the same wintering and breeding sites during autumn

migration (19 clusters, 64 individuals). Numbers in brackets depict individuals within the same cluster; different colours represent birds in the same breeding/wintering clusters. Dots: wintering sites; triangles: breeding sites. Source of basemap: Esri, HERE, Garmin, © OpenStreetMap contributors, and the GIS user community.

Supplement 5:

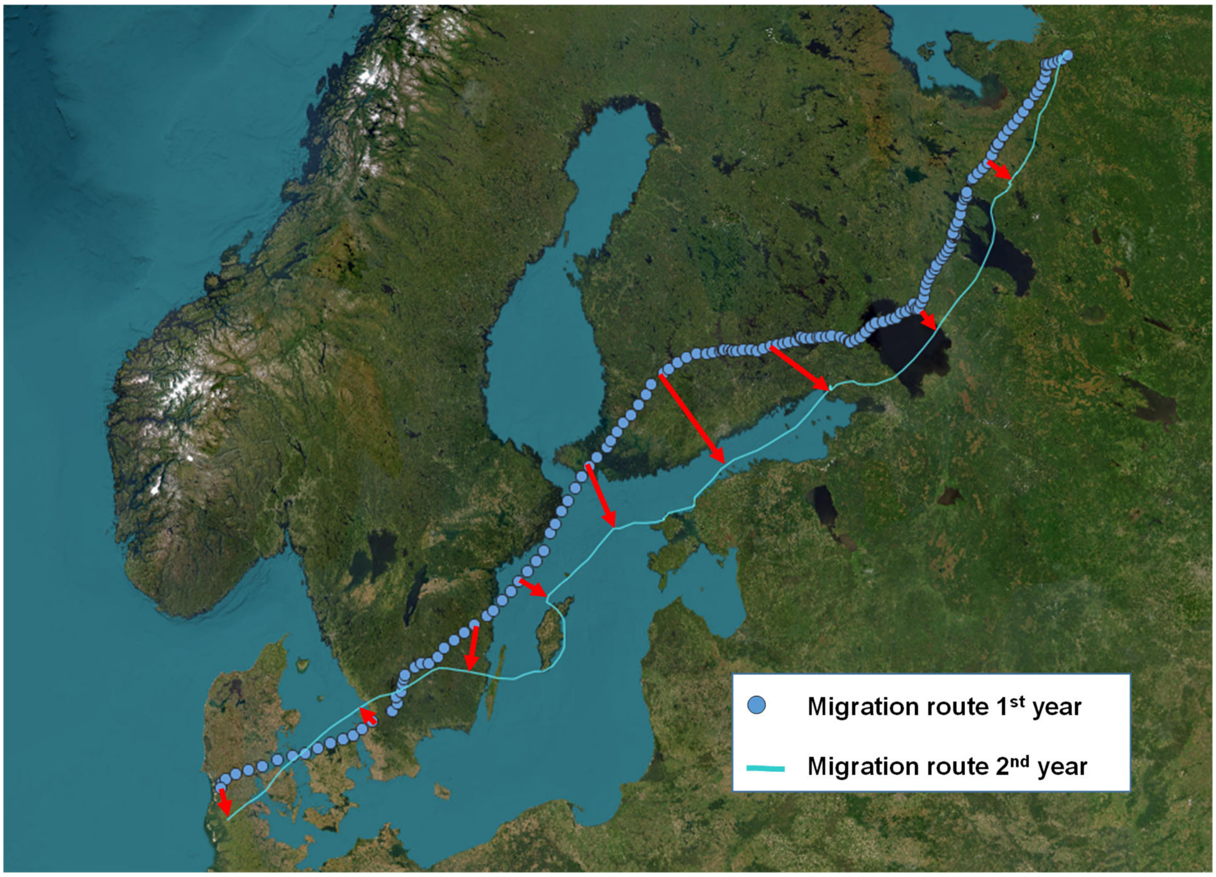

Figure S4: Assessment of distance between migration routes in consecutive years. The nearest distance of each GPS fix for the first year's track to the nearest neighbour of the next year's track for the same individual was computed. Overall means and overall medians of all nearest neighbour distances for consecutive tracks were computed to assess track similarity. Source of basemap: Esri, Vantor, Earthstar Geographics, and the GIS User Community.
