# Supplementary material for: High annual-cycle repeatability suggests low flexibility to environmental changes in a near-threatened migratory shorebird
Source: Commun Biol. 2026 May 30;9:736. doi: 10.1038/s42003-026-10371-0 (PMC13222360; doi:10.1038/s42003-026-10371-0)
Supplement: Supplementary file 2 — Description of Additional Supplementary Materials [file 42003_2026_10371_MOESM2_ESM.pdf]

Description of Additional Supplementary Files

File name: Supplementary Data 1

Description: the source data behind Fig 1 and Fig. 2 in the paper

File name: Supplementary Data 2

Description: the source data behind Fig. 3, Fig 4 and Fig. 5 in the paper

File name: Supplementary Data 3

Description: the source data behind Fig. 6 in the paper

File name: Supplementary Data 4

Description: the source data behind Fig. 7 in the paper

File name: Supplementary Data 5

Description: Overview of sample sizes and repeated samples used
